# Aging and sarcopenia associate with specific interactions between gut microbes, serum biomarkers and host physiology in rats

**DOI:** 10.18632/aging.101262

**Published:** 2017-07-17

**Authors:** Jay Siddharth, Anirikh Chakrabarti, Alice Pannérec, Sonia Karaz, Delphine Morin-Rivron, Mojgan Masoodi, Jerome N. Feige, Scott James Parkinson

**Affiliations:** ^1^ Nestlé Institute of Health Sciences SA, EPFL Innovation Park, 1015 Lausanne, Switzerland

**Keywords:** aging, microbiome, sarcopenia, lipidomics, proteomics, muscle physiology

## Abstract

The microbiome has been demonstrated to play an integral role in the maintenance of many aspects of health that are also associated with aging. In order to identify areas of potential exploration and intervention, we simultaneously characterized age-related alterations in gut microbiome, muscle physiology and serum proteomic and lipidomic profiles in aged rats to define an integrated signature of the aging phenotype. We demonstrate that aging skews the composition of the gut microbiome, in particular by altering the *Sutterella* to *Barneseilla* ratio, and alters the metabolic potential of intestinal bacteria. Age-related changes of the gut microbiome were associated with the physiological decline of musculoskeletal function, and with molecular markers of nutrient processing/availability, and inflammatory/immune status in aged versus adult rats. Altogether, our study highlights that aging leads to a complex interplay between the microbiome and host physiology, and provides candidate microbial species to target physical and metabolic decline during aging by modulating gut microbial ecology.

## INTRODUCTION

The concept of ‘healthy aging’ addresses the need to combat the economic burden of an aging population, its physiological and social consequences, and subsequent reduced quality of life. Microbes and multicellular organisms have co-evolved over millennia. The micro-bial counterpart has been shaped in terms of their composition and metabolic potential by taking cues from the state of their hosts’ physiology and immediate external environments [1]. Aging is characterized by alterations in distinct sets of host functions including cellular function (leading to oxidative stress and senescence) and a pathophysiological decline of most organs and metabolic homeostasis [2–4]. In particular, there is decline of the musculoskeletal system [5, 6], which contributes to alterations in the quality of life.

The gut microbiota may directly or indirectly impact several age-related aspects [7–12] and is an under-explored area of investigation [13, 14].

The microbiome has been implicated in several aspects associated with aging including rate of aging [15], inflammation [16], immunity [17], and muscle status [18]. Previously, it was shown that there is a significant relationship between age and the taxonomic and altered metabolic potential of the microbiome in mice [8]. Associations between microbiome, age and pro-inflammatory status (serum MCP-1 status) mice have also been identified [19]. However, an integrated view of age-related alterations in gut microbiome, muscle physiology, and biochemical protein and lipid markers would help to define areas of further investigation and potential intervention to support ‘healthy aging’.

Previously, we investigated the susceptibility of muscles in rats to age-related decline [20]. In the current study, we characterized the same rats to determine a metabolic fingerprint of the aging phenotype and investigated the associated alterations of the microbiome as a contributor to age-related physiology and sarcopenia. We identified age-specific features in the gut microbial communities for 8, 18 and 24-month rats and age-related alterations of the microbial metabolic potential. At the physiological level, we observed decreased gastrocnemius muscle mass and sciatic response amplitude that correlated with vitamin B12 levels and lipid metabolism. We identified several known and novel associations between gut microbiota and physiological parameters in aging. In summary, the data pointed towards changes in nutrient processing, musculoskeletal status and inflammatory/immune status with aging. This enabled us to define a consensus phenotype of age-related alterations in gut microbiome, muscle physiology, and biochemical protein and lipid markers of aging.

## RESULTS

**Figure 1:**
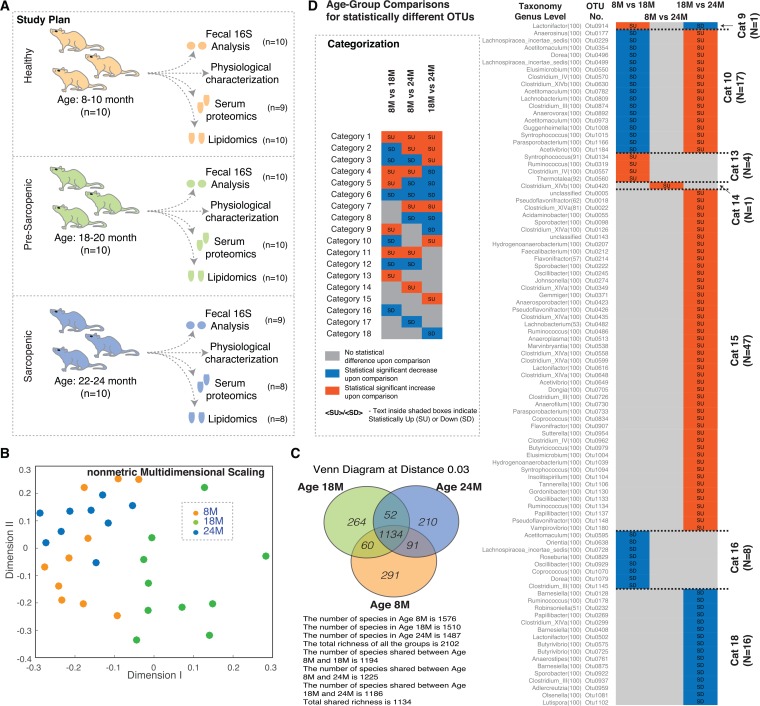
Gut microbial diversity in aging rats (**A**) Study design highlighting the experimental plan and the measured parameters. (**B**) NMDS plot of OTUs using Jclass calculator for the 16S data. The points show a distinct cloud for age group 18M (green circles), while ages 8M (orange circles) and 24M (blue circles) show more overlap. (**C**) Overlap of observed OTUs between the different age groups. (**D**) Comparison of statistically different OTUs across different age groups and classification into categories. Vignette: Categorization/feature based classification of members based on statistical increase/decrease across different age windows. Abbreviations: SU – Statistically Up, SD – Statistically Down, 8M – 8 months, 18M – 18 months and 24M – 24 months.

In this study we sought to investigate the association of age and sarcopenia (AAS)-related gut microbial changes with host physiology and identify the potential molecular mechanisms underlying these associations to evaluate the potential of targeting the microbiome in AAS-related health. Using aged Wistar rats of ages 8, 18 and 24 months (subsequently referred to as 8M for adult, 18M for adult-pre-sarcopenic and 24M for adult-sarcopenic respectively (similar framework as our previous work [20]), we determined the ecological states of the microbiome at the various ages, identified potential metabolic functions of these states and integrated this analysis with the biochemical and physiological phenotypes (Figure 1A).

### Gut microbial diversity in aged rats

The gut microbiome is a complex ecosystem that reflects the contribution of multiple environmental (such as diet, drugs and pathogens) and host-related (immunity) factors. We first sought to understand the composition of the microbiota across the ages to determine key bacteria associated with the aging phenotype.

We began by conducting a 16S rRNA-based OTU survey of the ecological state of the fecal microbiomes (Supplementary Materials, S1.xlsx). Our observations of the microbial ecology (using NMDS plots of the community structures Beta diversity) suggest that the microbiome is affected by aging. The 18M communities appeared unique to the 8M and 24M communities that could not be distinguished from one another (Figure 1B). The composition (alpha diversity) of the microbiome at the three ages demonstrated unique OTUs at each age, but generally did not distinguish one age-related community from another (Figure 1C).

The OTUs identified in the 16S survey were organised into categories based on the observed abundance across the ages (Figure 1D) (further described in Supplementary Materials). We observed 7 of the possible 18 categories (theoretically possible) that would demonstrate a significant change. In particular, categories 9 and 10 demonstrated the same pattern as seen in the NMDS plots identifying potential OTUs that drive the distinction of the 18M samples.

**Figure 2:**
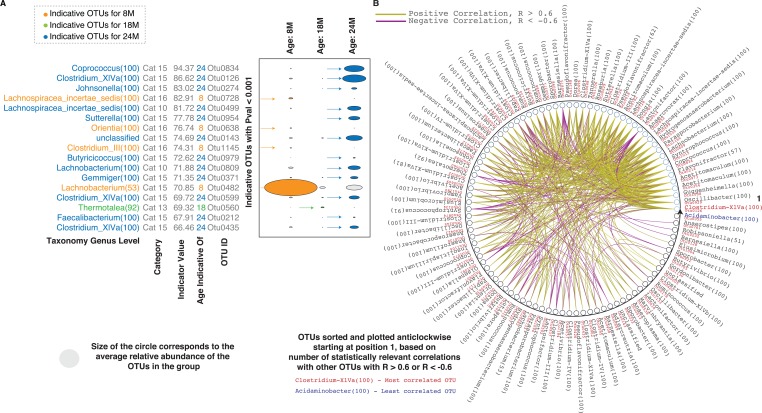
Inter-species correlations in the aging rat microbiome (**A**) Indicator analysis for different age groups. Indicator OTUs with P values < 0.001 for different age groups – orange for 8M, green for 18M and blue for 24M are shown. Categorization of the indicator OTUs and their corresponding indicator values are also indicated. (**B**) Correlations between the statistically different OTUs. Only correlations values > 0.6 and < -0.6 are shown. The OTUs are sorted and plotted anticlockwise starting at 0 degrees, based on the number of correlation (statistically relevant and with R values > 0.6 or < -0.6) across the entire set. The OTU classified as *Clostridium XIVa* (highlighted in red text) at the genus level is the most correlated while the OTU classified as *Acidaminobacter* (highlighted in blue text) is the least correlated. Details of one to one OTU correlations are in Supplementary Figure 1 and Supplementary Materials. Abbreviations: SU – Statistically Up, SD – Statistically Down, Cat – Category, 8M – 8 months, 18M – 18 months and 24M – 24 months.

We also used indicator analysis [21] to investigate the contributions of different OTUs to the differences between the age groups. Thirty-nine unique indicator OTUs with P values < 0.001 and statistically different levels upon comparison of two age groups were identified for ages 8M, 18M and 24M (Supplementary Materials, S1.xlsx–IndicatorAnalysis Sheet). Selected indicator OTUs (indicator values > 65), their average relative abundance, their categorization and the age group they indicate are depicted in Figure 2A. OTUs indicating 24M were the most abundant. The only indicator OTU for 18M, with an indicator value (69.32), was OTU0560 (*Thermotalea)* which was also significantly upregulated at 18M vs 8M. Focussing on the category 9 and 10 OTUs, OTU 0499 (*Lachnospiraceae insertae sedis*) and OTU 0809 (*Lachnobacterium*) were both identified as indicators of the 24M group, consistent with the observed decrease at 18M (Figure 1D). Based on Spearman correlations, these two OTUs were also some of the most connected OTUs based on their positive correlation with other OTUs across the ages (Figure 2B, Supplementary Figure 1). OTU 0914 (*Lactonifactor*), the only OTU found in category 9, was also well connected but negatively correlated with a number of OTUs including OTUs 0499 and 0809. Based on the composition of the microbes across the ages, these three OTUs appear to be markers and keystones of the 18M microbial ecology.

### Association of bacteria genera and rat sarcopenia

**Figure 3:**
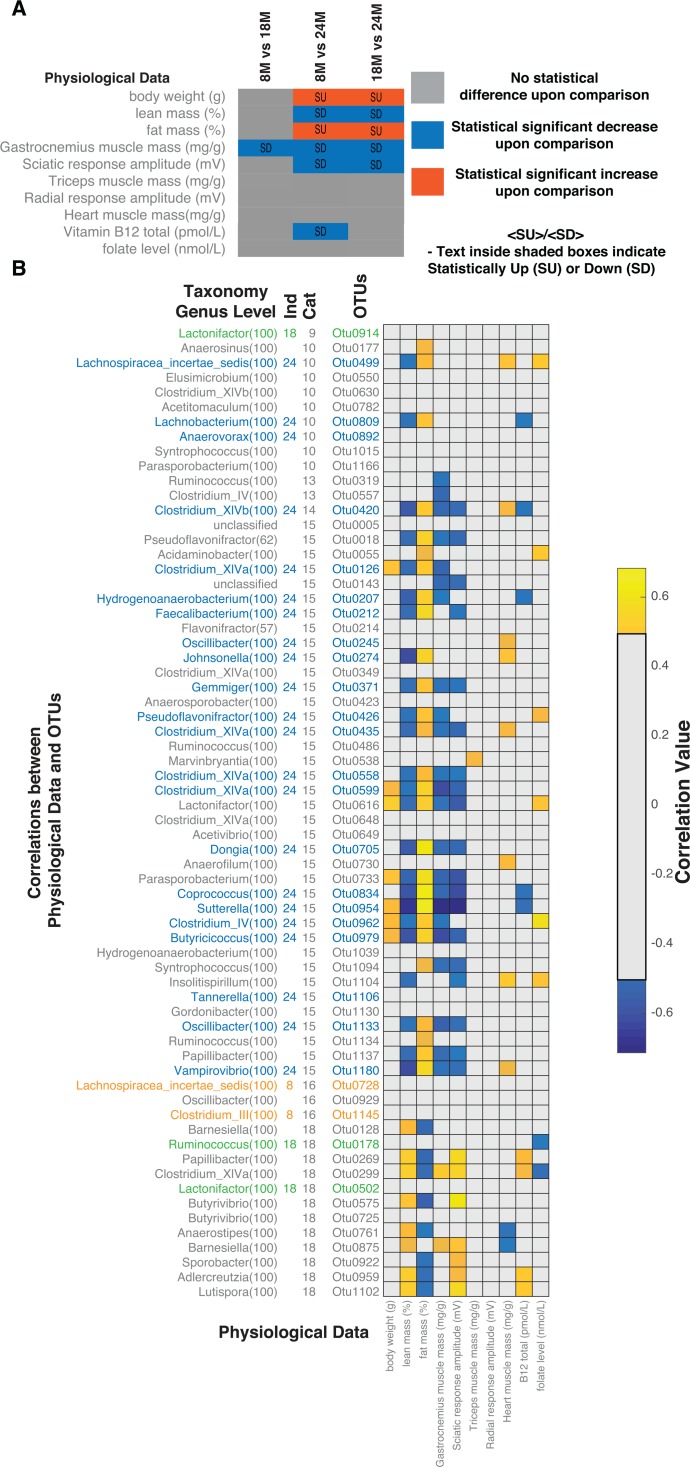
Correlations between microbiome and host physiology (**A**) Age group comparisons for statistical differences of measured physiological parameters, body weight (g), lean mass (%), fat mass (%), gastrocnemius muscle mass (mg/g), sciatic response amplitude (mV), triceps muscle mass (mg/g), radial response amplitude (mV), heart muscle mass (mg/g), Vitamin B12 total (pmol/L) and folate levels (nmol/L). (**B**) Correlations between statistically different OTUs and physiological measurements. Correlations shown are after FDR correction with Q values < 0.05. Abbreviations: SU – Statistically Up, SD – Statistically Down, Cat – Category, 8M – 8 months, 18M – 18 months and 24M – 24 months.

We next examined the association between the microbial ecology and the observed physiological changes in the rats. Aged rats gradually lose muscle mass and function (i.e. sarcopenia) through multi-factorial mechanisms involving mitochondrial and neuromuscular dysfunction [20, 22]. Having characterized the genetic composition of the microbiome, we investigated whether the age-related changes observed in the ecology of the gut microbiota could account for some of the physiological parameters associated with aging and sarcopenia. We previously demonstrated in the same rats that they demonstrated selective age-related sarcopenia (Table 1, complete data provided in Supplementary Materials S3.xlsx). Using age window comparisons of the physiological measurements we observed that body weight and fat mass (%) increased with age (statistically different in 8M-24M and 18M-24M comparisons) while gastrocnemius muscle mass (statistically different in 8M-18M, 8M-24M, 18M-24M comparisons), lean mass (%) and sciatic response amplitude (statistically different in 8M-24M and 18M-24M comparisons) decreased with age (Figure 3A). To investigate the potential associations between the microbiome and the host, we analysed the correlations (details in materials and methods) between the physiological measurements and the OTUs significantly correlated with age (Figure 3B). We focussed on the indicator OTUs for the 24M samples consistent with the development of the sarcopenia phenotype. In general, these OTUs (24M) correlated with increased fat mass, decreased lean and gastrocnemius muscle mass. Several of these OTUs also correlated with vitamin B12 and folate levels. The group 10 OTU (OTU0499) positively correlated with both folate levels in the plasma and heart mass. Although folate levels did not significantly change across the ages, its role in the context of cardiovascular disease has been studied extensively. Vitamin B12, as well as folate, have been similarly studied in the context of lipid physiology. Considering the association of these bacteria with these vitamins as well as the physiological impact of aging, we next sought to determine the underlying mechanistic links between the microbiota and host physiological responses to aging.

**Table 1:** Physiological parameters measured in aged rats. Std Dev – refers to standard deviation

Physiological Parameters	Average 8M	Std Dev 8M	Average 18M	Std Dev 18M	Average 24M	Std Dev 24M
Body weight (g)	518.72	52.82	555.50	36.20	606.92	37.69
Lean mass (%)	72.75	2.12	71.21	2.34	66.88	1.44
Fat mass (%)	12.54	2.43	13.32	2.42	18.58	1.76
Gastrocnemius muscle mass (mg/g)	4.84	0.76	3.92	0.55	2.32	0.39
Sciatic response amplitude (mV)	65.86	14.88	60.26	15.18	26.66	11.63
Triceps muscle mass (mg/g)	3.88	0.67	3.92	0.23	3.81	0.35
Radial response amplitude (mV)	69.32	14.79	70.96	11.61	63.51	17.03
Heart muscle mass (mg/g)	2.71	0.46	2.74	0.30	3.07	0.45
B12 total (pmol/L)	1074.00	183.97	965.00	167.10	912.50	258.94
Folate level (nmol/L)	169.05	27.83	170.75	24.18	205.50	47.08

### Metagenomic functional content analysis

**Figure 4:**
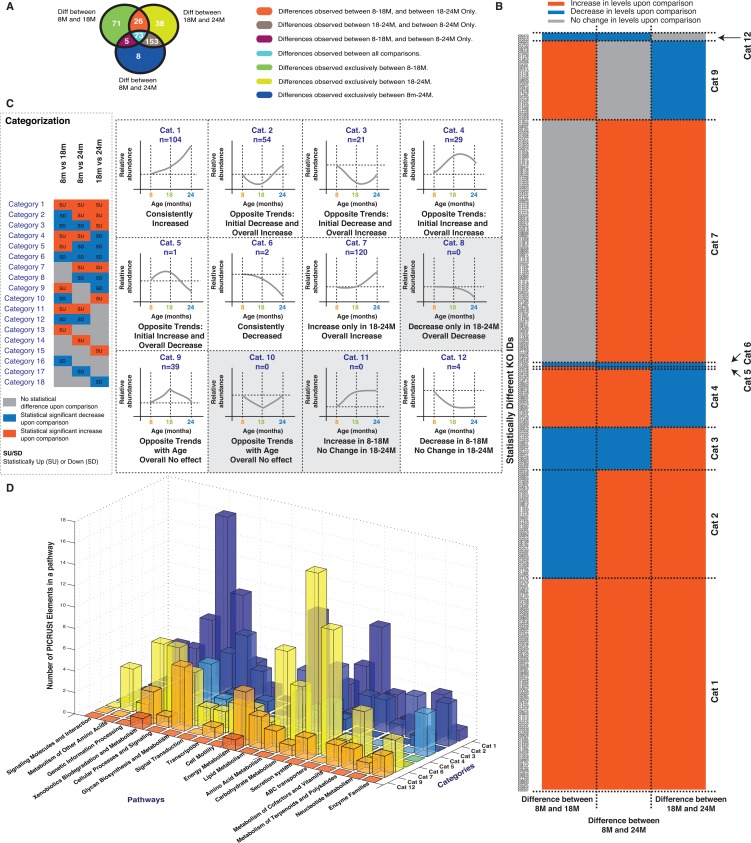
Analysis of predicted Metagenomic Functional Content (MFC) obtained from PICRUSt (**A**) Statistically different MFC's for each comparison (8M vs 18M, 8M vs 24M, 18M vs 24M) and their overlaps are depicted. (**B**) Comparison of the cumulatively unique statistically different MFC's identified in panel A is depicted across each of the studied comparisons. Red shaded boxes indicates increase in MFC levels, Grey boxes indicate no statistical difference and Blue boxes indicates decrease in MFC levels. The MFC's are sorted according to different categories as explained in panel C. (**C**) Explanation of the categories or feature based classes for the statistically different MFC's. (**D**) Membership of the statistically different MFC's in each category in different pathways. Correlations shown are after FDR correction with Q values < 0.05. Abbreviations: SU – Statistically Up, SD – Statistically Down, Cat – Category, 8M – 8 months, 18M – 18 months and 24M – 24 months.

We next tried to identify potential molecular mechanisms implicated in the aging phenotype by applying PICRUSt [23] to identify the Metagenomic Functional Content (MFC) for different groups using the 16S rRNA data (Figure 4A). The tabulated entries for the entire list of statistically different MFC's are available in the Supplementary Materials (Supplementary Materials, S2.xlsx).

To help focus the analysis, the MFCs were put into the same 18 categories previously used for the OTUs in Figure 1 (Figure 4B, 4C and Figure 1D). All the statistically different MFC's could be classified into 9 categories, with the majority (82% or 307 KO IDs, categorised into categories 1, 2, 4 and 7). Most of the physiological responses (Figure 3A) could be categorised into Category 7 or 8 (significantly higher/lower at the 24M vs 8/18M). Since there were no MFCs observed in category 8, we focussed our attention on the Category 7 observations. These can be summarised in three main categories: secretion systems (often associated with pathogenic mechanisms), ABC transporters (often associated with uptake of essential nutrients) and dietary metabolism (protein, carbohydrate and lipid metabolism) suggesting that the host/micro-biome interactions contributing to the aging phenotype involves immune and dietary components.

Microbial NMDS analysis and the community analysis summarised in Figures 1 and 2 indicated that the 18M microbiota was distinct from the 8M and 24M communities. While the composition of the 8M and 24M communities could not be distinguished based on 16S rRNA survey, their functional capacity was distinct as demonstrated in Figure 4. One interpretation of this is that there was a transition in the community membership that led to a functional alteration of the microbiota. The 18M community MFC therefore may give an indication of the molecular mechanisms that underlie this transition. Figure 1 identified Category 9 and 10 OTUs as associated with the 18M samples. While no category 10 MFCs were observed, there were an abundance of category 9 MFCs. We further summarized the MFC's into their corresponding KEGG pathways to hypothesize the molecular mechanisms that may underlie the transition to the aged phenotype (Figure 4D). Category 9 pathways were primarily related to diet including metabolism of carbohydrate, protein, lipids and vitamin biosynthesis (Supplementary Materials, S2.xlsx) suggesting that the microbiome contributes in part to aging through their established role in digestion. We observed a general increase in MFC's assigned to Amino Acid (25/29) and Carbohydrate (12/15) metabolism with a concurrent general decrease (6/9) in those assigned to Lipid Metabolism suggesting a shift in the potential to digest, process, and synthesize these dietary components. One of the increased MFCs was cholesterol oxidase (steroid biosynthesis) indicating a predicted change towards cholesterol versus fatty acid synthesis.

Furthermore, consistent with the analysis of MFCs related to Metabolism of Cofactors and Vitamins (Figure 4D), statistically reduced levels of serum Vitamin B12 were observed. We also explored the correlations between specific MFC's and the physiological measurements (Table 1, Supplemental Figure 2). None of the Category 9 MFCs associated with physiological parameters. However, there were several associations of other diet-related MFCs on many physiological factors including gastrocnemius muscle mass and sciatic response amplitude (Supplementary Figure 2, S2.xlsx – MFC_Metadata_Correlation Sheet). Fat mass was positively correlated to Cobalamin biosynthetic protein CobC (K02225), carnitinyl-CoA dehydratase (K08299) and allantoin permease (K10975). Furthermore, there was a strong negative correlation between lean mass and allantoin permease (K10975). Allantoin has previously been demonstrated to increase lifespan when administered to the nematode worm *Caenorhabditis elegans* [24, 25]. The negative association could relate to microbial scavenging of this purine metabolism biomarker of oxidative stress [26].

We considered the following key observations: 1) analysis of the aging microbiome identified a diet-related ecology specifically associated with the transition at 18M to the sarcopenic phenotype, 2) the OTUs characterized at the 18M correlated with observed Vitamin B12 and folate levels, and 3) the MFC analysis identified candidate molecular mechanisms including pathogenesis and dietary metabolic function associated with the sarcopenic phenotype. Taking these observations together, they indicate an important role for microbial-derived dietary metabolic pathways in aging and sarcopenia including carbohydrate, lipid and vitamin metabolism that could alter the metabolic status of the host and contribute to the physiological state in aging.

### Serum proteomics of aged rats

**Figure 5:**
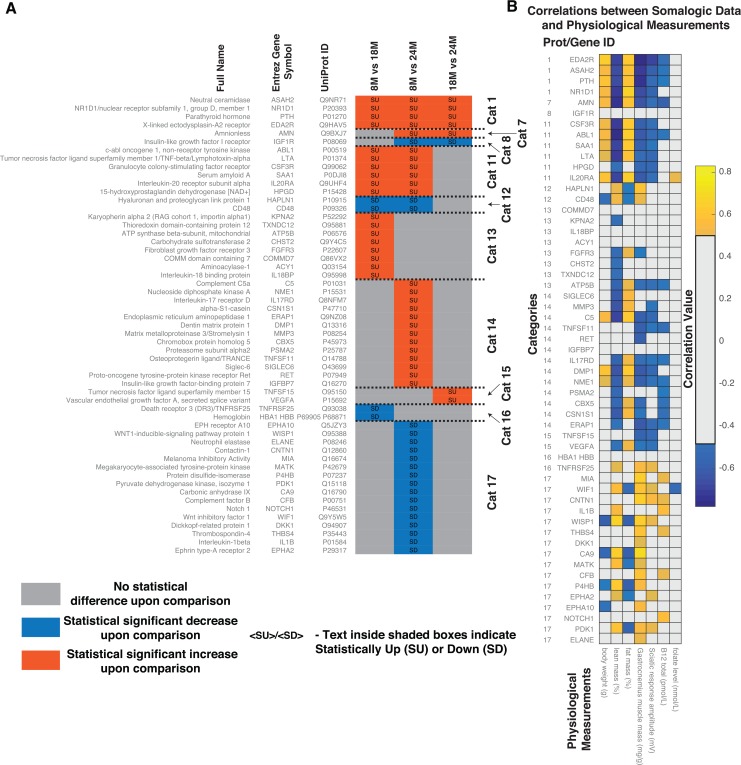
Comparative analysis of proteomics data from the serum of aging rats (8M, 18M and 24M) obtained using aptamer-based detection method (**A**) Based on the pattern of statistically significant increase (SU) and statistically significant decrease (SD) between the different ages, the proteins were classified into categories. Protein full names, Entrez Gene Names, UniProt IDs and corresponding categorical classifications of the statistically different proteins identified in the serum of the aging rats. Abbreviations: SU – Statistically Up, SD – Statistically Down, Cat – Category, 8M – 8 months, 18M – 18 months and 24M – 24 months. (**B**) Correlations between statistically different serum proteins and physiological measurements. Correlations shown are after FDR correction with Q values < 0.05.

As shown by the analysis above, microbial ecology is a complex process that involves the integration of many factors leading to an outcome in the host. We performed proteomic analysis of the serum to identify the potential host response to the changes in the microbiota and their association with the physiological state. Serum proteins for the 3 groups of rats (n=9 at 8M, n=10 at 18M and n=8 at 24M) were analysed using aptamer-based detection [27]. Similar to the OTUs and MFC analysis, we performed pairwise comparisons to identify proteins that were significantly altered between two age groups (8M-18M, 8M-24M and 18M-24M) and put them into the categories previously described. (Figure 5A, Supplementary Materials S4.xlsx).

Since we were looking specifically at the host response, we focussed on categories that demonstrated a significant association with the host physiological measurements shown in Figure 3. In general, fat mass, lean mass and sciatic response amplitude demonstrated a ‘Category 7 or 8′ pattern. There were only two serum proteins detected demonstrating this pattern; Amnionless (AMN) and Insulin like growth factor I receptor (IGF1R). IGF1R did not show any significant correlation with the physiological measurements. However, AMN correlated with most of the physiological measurements with the exception of folate (Figure 5B). AMN plays a key role in Cobalamin (Cbl, vitamin B12) transport as well as for lipid metabolism, HDL-mediated lipid transport, lipoprotein metabolism and lipid digestion. We also detected an association of AMN with cholesterol oxidase and cobalamin bio-synthetic protein CobC in the MFCs (Supplementary Figure 4).

Subsequently, we used Reactome [28, 29] (www.Reactome.org) to identify participation of the differentially observed proteins in different pathways in rats (Supplementary Materials S4.xlsx). Overall, protein levels which consistently increased with age (Cat 1), i.e. Neutral ceramidase (ASAH2), Nuclear receptor subfamily 1 group D member 1 (NR1D1), Parathyroid hormone (PTH) and X-linked ectodysplasin-A2 receptor (EDA2R) were implicated in glycosphingolipid metabolism, sphingolipid metabolism, GPCR signalling, TNFR2 non-canonical NF-kB signalling and the immune system.

Summarizing the genetic analysis of the microbiome and the serum protein response of the host there was a common theme associated with the microbiome and aging. Alterations in dietary metabolism, possibly via their influence on Vitamin B12 and folate, seemed to be involved in the aging phenotype.

### Serum lipidomics of aged rats

**Figure 6:**
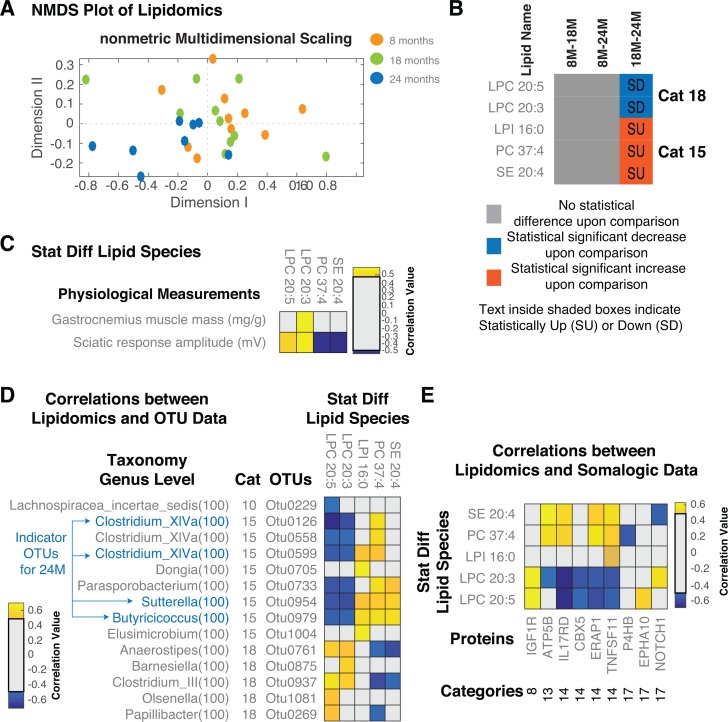
Serum Lipidomic analysis of aged rats (**A**) NMDS plot of the lipid species measured across all the samples. Overall, we see a separation between 18M and 24M but not between 8M-18M and 8M-24M. (**B**) Statistically different lipid species and demarcation of up/downregulation in different comparisons. (**C**) Correlations between lipid species and measured physiological parameters. (**D**) Correlations between lipid species and OTUs. (**E**) Correlations between lipid species and proteins. Only statistically significant correlations are shown. Correlations shown are after FDR correction with Q values < 0.05. Abbreviations: SU – Statistically Up, SD – Statistically Down, Cat – Category, 8M – 8 months, 18M – 18 months and 24M – 24 months.

Vitamin B12 and folate have defined roles in lipid metabolism. Vitamin B12, adenosylcobalamin, act as a cofactor on methylmalonyl-CoA mutase to convert methylmalonyl-CoA (MM-CoA) to succinyl-CoA. When MM-CoA mutase is blocked in the absence of Vitamin B12, MM-CoA accumulates and inhibits the rate-limiting enzyme of fatty acid oxidation (CPT1 - carnitine palmitoyl transferase) thus causing lipo-genesis. Low levels of Vitamin B12 are also associated with shifts toward cholesterol synthesis and hepatic steatosis in mice [30]. Folate deficiency decreases flux through phosphatidylethanolamine *N*-methyltransferase (PEMT), an enzyme that synthesizes phosphatidyl-choline (PC) via the methylation of phosphatidylethano-lamine (PE) [31]. The alterations in these lipid-modifying vitamins by the microbiota therefore could underlie the association with aging. We analysed 122 lipid species using direct infusion mass spectrometry as described previously [32] (materials and methods, data tabulated in Supplementary Materials, S5.xlsx). Analysing the measured lipid species across the samples (n=10 at 8M, n=10 at 18M and n=8 at 24M), using NMDS analysis, there was no clear separation between 8M and 18M (orange and green circles in Figure 6A).

One to one comparison for statistical differences between two groups further confirmed this (no statistically different levels for lipid species measured between 8M and 18M or between 8M and 24M). However, the 18M and 24M groups clearly separated in the lipid space (green and blue circles, Figure 6A). 5 lipid species were statistically significantly different between the two groups (18M-24M) (Figure 6B). Levels of two Lysophosphatidylcholines (LPC 20:5 and LPC 20:3) decreased in old age, while three lipids, Lysophosphatidylinositol (LPI 16:0), Phosphatidyl-choline (PC 37:4) and sterol ester (SE 20:4) increased in old age. The changes in phosphatidylcholine/lyso-phosphatidylcholine are consistent with the observed alterations in Vitamin B12 and folate status. Subsequently, we looked at the correlations of the lipids with physiological measurements (Figure 6C). Gastrocnemius muscle mass (decreased consistently with age) and sciatic response amplitude (decreased in 8M-24M and 18M-24M) significantly correlated to the statistically different lipids (Figure 6C). Gastrocnemius muscle mass positively correlated with LPC 20:3 (decreased in 18m-24m). Sciatic response amplitude positively correlated with LPC 20:5 and LPC 20:3 (both decreased in 18M-24M) and negatively correlated with PC 37:4 and SE 20:4 (both increased in 18M-24M) (Figure 6C). Lipid metabolism, therefore, may play a particular role in the development of the sarcopenic phenotype observed in these rats.

Given the possible role of microbes in altering lipid and inflammatory status, we looked at the correlations of the lipid species with the microbial members (OTUs – Figure 6D, MFC's – Supplementary Figure 5) and serum protein levels (Figure 6E). In total there were 14 OTUs that correlated with the age-related lipid species of which OTU 0229 (*Lachnospiraceae insertae sedis*) was the only category 10 OTU identified. In particular, it negatively correlated with LPC 20:5 (Figure 6D). The other OTUs identified were, similar to the lipids identified, Category 15 and 18 OTUs. Of these, OTUs 0126, 0954, 0979 and 0599 were also identified in the indicator analysis in Figure 2. Focussing on these, OTU 0954 also negatively correlated with Vitamin B12 levels (Figure 3). The category 18 OTU 0269 was also idenified and positively correlated with B12 (Figure 3). LPC 20:5, LPC 20:3 and PC 37:4 were among the most correlated lipid species consistent with a potential role for microbial metabolism of Vitamin B12 and folate. In addition, the levels of PC 37:4 and SE 20:4 positively correlated with the MFC Cobalamin Biosynthesis Protein CobW (Supplementary Figure 5). Reduced LPC 20:5 were reported in obese patients as opposed to control subjects [33]. LPC 20:5 and LPC 20:3 were earlier reported to be negatively correlated to BMI in Obesity [34]. However, the association of these lipids with a sarcopenic phenotype suggests an important role for the microbiome in regulating lipid metabolism, potentially contributing to sarcopenia as well as obesity.

We next determined potential host responses to the altered lipid physiology. We identified 9 proteins, which are significantly correlated with the lipid levels. Proteins, which are the most correlated, included IL17RD, ERAP1, TNFSF11 and ATP5B. While the lipids, which were the most correlated, included LPC 20:3, LPC 20:5, PC 37:4 and SE 20:4. Reduction of NOTCH1 was negatively correlated to SE 20:4 and positively correlated to LPC 20:3. Potential anti-inflammatory lipids LPC 20:5 and LPC 20:3 were negatively correlated to increased levels of IL17RD, CBX5, ERAP1 and TNFSF11 and positively correlated to IGF1R. Alterations of the EPH-ephrin signalling (via EPHA10) was positively correlated to LPC 20:5. Proteins altering the immune status (i.e. TNFSF11, ERAP1 and IL17RD) were negatively correlated to decreased levels of LPC 20:5 and LPC 20:3 and positively correlated to increased levels of PC 37:4 and SE 20:4. The negative correlation of P4HB to PC 37:4 levels indicates potential effects in VLDL biosynthesis and chylomicron-mediated lipid transport.

Our results demonstrate the complexity of host microbe interactions in the context of aging and highlight the potential interaction of nutrition, the microbiome, and host metabolism in sarcopenia. Our thorough survey of microbial, proteomic and lipidomic biomarkers that underlie the aging process suggest that regulation of dietary metabolism by the microbiota may underlie some of the consequences of aging and identify new nutritional therapeutic avenues for further exploration in age-related conditions such as sarcopenia.

## DISCUSSION

Microbial association with its host has been shaped over millennia. The ecosystem is in a state of constant remodelling adapting to its host environment [35–39]. This in turn impacts host function, including at the extremes of aging [3]. Researchers [40] have observed that the microbial membership changes as the host ages, although the molecular mechanisms underlying the association of aging and microbial ecology are yet to be defined. Combinations of several factors including but not limited to clinical status, prevalence of co-morbid diseases, exposure to multiple medications, aging alimentary tracts, impaired dentition, decreased gut motility and dietary modifications complicate investigation in humans. However, some aspects like inflammation [41], muscle [4, 11, 20, 42–44], bone [12, 45] and immune status [46] have been previously investigated. It is thus relevant and important to understand the complex associations between gut microbes and host physiology to determine the scope of the potential of lifestyle/nutrition/pharmacological interventions to maintain health in old age.

**Figure 7:**
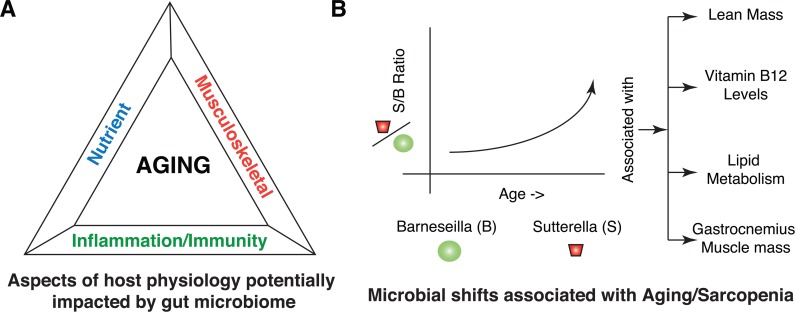
Summary of host-microbial interactions in Aging and Sarcopenia (**A**) Different aspects of host physiology impacted directly or indirectly by gut microbiome include nutrients, musculoskeletal and inflammation/immunity. (**B**) Illustration highlighting the shift within the microbial community in terms of members of *Sutterella* and *Barnesiella* with aging. *Barnesiella* is positively correlated to *Clostridium XIVa* and *Papillibacter*, which are all similarly correlated to aging phenotypes, specifically at the level of Lean Mass, Vitamin B12 levels, Lipid metabolism and Gastrocnemius muscle mass.

Our systematic integrated analysis of *in-vivo* phenotyping, gut microbial analysis, biochemical analysis, serum proteomics and lipidomics of aged sarcopenic rats (8M, 18M and 24M) highlighted three broad associations with aging; musculoskeletal, nutrient and inflammation/immunity (Figure 7A). Musculo-skeletal modifications broadly included altered muscle function and serum markers associated with altered bone density and bone regeneration. Modifications in nutrient availability/role broadly included altered vitamin production/uptake, altered carbohydrate metabolism and altered lipid metabolism/uptake. Inflammation/Immunity related changes broadly included chronic/age specific inflammation, altered immune status, impact on T cells and altered mucin layers in the gut.

Modifications in muscle status with aging has been extensively studied and discussed in several studies [4, 11, 20, 42, 43]. Consistent with earlier findings, at the physiological level, we observed decreased gastro-cnemius muscle mass and decreased sciatic response amplitude with aging. Our integrated analysis suggests that the microbiota may underlie the sarcopenic phenotype of the aged rats via vitamin synthesis, altered lipid metabolism and regulation of growth and immune-related factors.

In particular, *Sutterella* (OTU0954) may have an important role in regulating some of the aspects of aging. Its presence correlated with AMN/vitamin B12, alterations in lipid metabolism as well as loss of gastro-cnemius muscle mass and sciatic response amplitude. Physiological decrease in serum Vitamin B12 levels were also consistent with altered metagenomic functional shifts in the microbes for cobalamin protein synthesis (CobC and CobW) and serum protein level changes in amnionless (AMN).

The ecology of the microbiome may play an important part in regulating this. While *Sutterella* positively correlated with the abundance of a number of indicator species (Figure 2), *Barnesiella* (OTU0875) negatively correlated with *Sutterella* and many of the same indicator species. In addition, it correlated positively with *Clostridium XIVa* (OTU0299) and *Papillibacter* (OTU0269). Individually, these three OTUs generally demonstrated the opposite profiles on the physiological parameters and the host proteomic response than *Sutterella*.

We therefore propose a model whereby *Sutterella* solidifies a microbial ecology that contributes to the aged phenotype in rats. This counteracts the positive influence of the *Barnesiella* on lean mass, alters Vitamin B12 and lipid metabolism, and results in a pro-inflammatory environment contributing to the sarcopenic phenotype (Figure 7B). Further studies will clarify whether this ecology is unique to rats, how it can be influenced by diet and whether these molecular mechanisms play a role in sarcopenia in human populations. Wang et al. [47] recently showed that probiotic strains like *Lactobacillus paracasei CNCM I-4270* (LC), *L. rhamnosus I-3690* (LR) and *Bifidobacterium animalis subsp. lactis I-2494* (BA), when administered to HFD fed mice, were potent in increasing OTUs affiliated to *Barnesiella*. While these authors tested the ability of these strains to reduce HFD-induced MS, it is interesting to hypothesize a potential role in preserving a healthy gut microbial ecology during aging and in delaying the pathological manifestations of aging. However, subsequent studies with detailed controls and establishment of causality will be required as next steps.

While the current study allowed us to define a consensus phenotype of aging and sarcopenia in rats, understanding the translational relevance and identifying interventional solutions will require further studies accounting for the differences between rat and human physiology, the gut microbiome [48, 49], and the interactions at play. Towards that goal, analysing human microbial ecology during aging and sarcopenia will be of major importance and transplanting these aged human microbiomes to germ free rodents will be key to uncouple aging of the microbiome from other physiological and environmental perturbations that co-exist during human aging.

## CONCLUSIONS

Considering the needs of the elderly in society and for cost-effective means to support healthy aging, our research findings are the most comprehensive characterization of the gut microbiome in context of the molecular mechanisms of aging in preclinical models studied to date. In our study, we simultaneously characterised and investigated age-related alterations in gut microbiome, muscle physiology and serum protein and lipid markers to define a consensus phenotype of age-related alterations in gut microbiome and host physiology. Interestingly, we observed changes in the composition and metabolic potential of the aging gut microbiome were associated with the musculoskeletal, nutrient processing/availability, and the inflammatory/immune status of aged vs adult rats. This study is the first step towards identifying the molecular mechanisms underlying microbiome-driven aging and sarcopenia and potential therapeutic interventions and guidelines to support healthy aging.

## MATERIALS AND METHODS

### Animal study protocol

Male Wistar rats aged 8 months to 24 months were obtained from Janvier Labs (Le Genest-Saint-Isle, France). The rats (littermates) labelled by age were grouped by date of birth within one month, and further grouping was then based on the muscle phenotype in hind limbs under the following categories: adult (8 months of age), early-sarcopenic (18 months of age), and sarcopenic (24 months of age). Upon arrival, all animals were housed by two in standard type 4 cages with ad libitum access to food and water on a 12 hour light/dark cycle at a temperature between 20-24°C and a relative humidity between 50-60%. The experimental procedures on animals were in agreement with Swiss and EU ethical guidelines and approved by the local animal experimentation committee of the Canton of Vaud (license number VD2630). Body composition (lean mass and fat mass) was measured non-invasively in awake animals using quantitative NMR (1H NMR relaxometry device, echoMRI). Electromyography (EMG) measurements were performed on rats under isoflurane anaesthesia. Briefly the left limbs were shaved and recording needle electrodes (twisted pairs, wire 150cm, needle 0.4 × 13mm, Neurolite, Switzerland) were sequentially placed into the gastrocnemius, and triceps brachii muscles. Supra-maximal electric stimulation was achieved via stimulating needle electrodes sequentially placed around the sciatic nerve and the radial nerve, and the resulting compound muscle action potential (CMAP) was recorded using the Keypoint software (Neurolite, Switzerland). The animals were sacrificed by exsanguination under isoflurane anaesthesia and blood was collected from the portal vein and resulting serum was used for proteomic analysis. Skeletal muscles and heart were dissected free of fat, weighed and snap frozen in liquid nitrogen.

### Fecal pellet collection, DNA extraction

Fecal pellets from each rat were collected prior to sacrifice in sterile 1.5 ml Eppendorf tubes that were then stored at -80C until DNA was extracted from approximately 50 mg using the MoBio PowerMag DNA extraction kit according to the manufacturer's specifications. The DNA was stored at -20C until library preparation.

### Amplicon library preparation and sequencing

An Amplicon library of partial 16s rRNA genes was produced according the methods outlined in Caparaso et al. [50]. Briefly, we used primers targeting the V4-6 region of the 16S ribosomal genes at positions 515-806 (*E. coli* numbering) composed of the Illumina adapters, pad, barcode (forward primer only) and gene specific nucleotides. The resulting PCR products after 23 cycles of amplification from 3 reactions per samples were pooled and quantified using a Caliper LabChip GX. The samples were then pooled in equimolar ratio and cleaned using Agencourt AMPure XP. The cleaned amplicons were then pooled with PhiX DNA spike as per manufacturer recommendation and sequenced using a MiSeq using chemistry V2.4.

### 16S data processing

Sequences were demultiplexed from fastq files according to the specific barcode for each sample introduced at the PCR step. Quality control was performed with MOTHUR [51] using the default parameters in the MiSeq SOP [52] accessed on Feb 2016. The resulting sequences were then clustered into OTUs (with 0.03 cutoff). These were also converted into biom files with closed OTU picking with greengenes taxonomy (greengenes version 13.5) for use in PICRUSt [53]. The resultant OTUs were also used for plotting NMDS with JClass and ThetaYC calculators, additionally an indicator analysis was also performed. Statistical differences in OTUs between age groups were assessed using the Wilcoxon Rank Sum test and after FDR correction, Q-values < 0.05 were considered significant.

### Metagenomic functional content analysis

Phylogenetic investigation of communities by reconstruction of unobserved states (PICRUSt) [53] was used to predict the metagenomic functional content (MFC) of the different samples. Statistical differences between age groups were estimated by using the Kolmogorov-Smirnov test on the rank of the z-scores of the relative abundances of the MFC's and P-values were considered significant using a FDR of 1%.

### Serum analysis

Vitamin B12 and folate levels were measured using the competitive binding assay from Beckman (Beckman Coulter, Nyon, Switzerland). For protein measurement in serum, samples were analyzed using DNA-aptamer-based recognition on the SOMAscan platform (SomaLogic, Boulder, CO, USA), as described [27]. Median normalized relative fluorescence units (RFUs) were log_2_-transformed before applying principal component analysis and linear models. Statistical analyses were performed in R 3.1.3 (R Foundation for Statistical Computing).

### Lipidomics analysis

Lipid extraction and analysis were performed as reported previously [32]. In summary, 100 ul of (diluted serum 1 in 50 ammonium bicarbonate buffer) was mixed with 80 ul of ammonium bicarbonate solution and then 810 ul of methyl tert-butyl ether/methanol (7:2, v/v) solution was added. Internal standard mixture was pre-mixed with the organic solvents mixture. The internal standard mixture contained: PC 17:0/17:0, Chol D6, DAG 17:0/17:0, TAG 17:0/17:0/17:0, Cer 18:1;2/17:0, SM 18:1;2/12:0, LPC 12:0, LPE 17:1, PE 17:0/17:0, SE 20:0, PI 16:0/16:0. Solution was mixed at 700 rpm, 15 min at 4°C using a ThermoMixer C and then centrifuged at 3000 RCF for 5 min. 100 μl of the organic phase was transferred to a 96-well plate, and dried in a speed vacuum concentrator. Lipid extract was reconstituted in 40 μl of 7.5 mM ammonium acetate in chloroform/methanol/propanol (1:2:4, V/V/V). All liquid handling steps were performed using Hamilton STAR robotic platform.

For MS data acquisition, samples were analyzed by direct infusion in a QExactive mass spectrometer (Thermo Fisher Scientific) equipped with a TriVersa NanoMate ion source (Advion Biosciences). Samples were acquired in both polarity modes in a single acquisition at Rm/z=200 = 140000. All data was analyzed with in-house developed lipid identification software based on LipidXplorer. Data post-processing and normalization were performed on an in-house developed R based package. Statistical significance between age groups was assessed using the Kolmogorov-Smirnov 2 sample test and after FDR correction Q-Values were considered significant if < 0.05.

### Correlation analysis

In general (unless otherwise stated), correlations (Spearman's Rho) were assessed between different measurements and checked for statistical significance. It was considered as statistical significant after FDR correction with Q values < 0.05.

### Ethics approval and consent to participate

Animal Study: The experimental procedures on animals were in agreement with Swiss and EU ethical guidelines and approved by the local animal experimentation committee of the Canton of Vaud (license number VD2630).

## SUPPLEMENTARY MATERIAL FIGURES













## Abbreviations

SCFA: Short Chain Fatty Acids
LPS: Lipo-polysaccharides
PICRUSt: Phylogenetic Investigation of Communities by Reconstruction of Unobserved States
8M: 8 month old rats
18M: 18 month old rats
24M: 24 month old rats
NMDS: Non-metric multi-dimensional scaling
OTU: Operational taxonomic unit
MFC: Metagenomic Functional Content
KO ID: KEGG Orthology ID
LPC: Lysophosphatidylcholine
LPI: Lysophosphatidylinositol
PC: Phosphatidylcholine
SE: Sterol Esters.
