# Supplementary material for: Aging and sarcopenia associate with specific interactions between gut microbes, serum biomarkers and host physiology in rats
Source: Aging (Albany NY). 2017 Jul 17;9(7):1698–714. doi: 10.18632/aging.101262 (PMC5559170; doi:10.18632/aging.101262)
Supplement: Supplementary file 1 [file aging-09-1698-s001.pdf]

SUPPLEMENTARY MATERIAL

Categorization or feature based classes

To analyse temporal features, we pooled the potential differences (statistically increased or decreased levels) between different age group comparisons, i.e. 8M-18M, 8M-24M and 18M-24M, into 18 theoretical categories (feature based classes, Figure 1D – Categorization Vignette). These categories correspond to: 1) continuous increase with age, 2) opposite trend with age: decrease between 8-18M, increase between 18-24M with overall increase between 8-24M, 3) opposite trend with age: decrease between 8-18M but increase between 18-24M with overall decrease between 8-24M, 4) opposite trend with age: increase between 8-18M but decrease between 18-24M with overall increase between 8-24M, 5) opposite trend with age: increase between 8-18M but decrease between 18-24M with overall decrease between 8-24M, 6) continuous decrease with age, 7) increase only between 18-24M and overall increase between 8-24M, 8) decrease only between 18-24M and overall decrease between 8-24M, 9) opposite trend with age: increase between 8-18M but decrease between 18-24M with overall no difference between 8-24M, 10) opposite trend with age: decrease between 8-18M but increase between 18-24M with overall no difference between 8-24M, 11) increase between 8-18M and no change between 18-24M with overall increase between 8-24M, 12) decrease only between 8-18M and no change between 18-24M with overall decrease between 8-24M, 13) increase only between 8-18M but no difference between 18-24M and 8-24M, 14) increase overall between 8-24M but no difference between 8-18M and 18-24M, 15) increase only between 18-24M but no difference between 8-18M and 8-24M, 16) decrease only between 8-18m but no difference between 18-24M and 8-24M, 17) decrease overall between 8-24M but no difference between 8-18M and 18-24M, 18) decrease only between 18-24M but no difference between 8-18M and 8-24M.

Availability of data and materials

1. The data including the raw sequences for the fecal samples analysed in the study reported in the paper has been deposited in the SRA database under the accession number **SRP092598**. Processed microbial data is provided in Supplementary Excel Sheets *S1.xlsx* and *S2.xlsx* - Data Set S1 and Data Set S2

2. The Physiological Data is provided in Table 1 and Supplementary Excel Sheet *S3.xlsx* - Data Set S3.

3. The Somalogic (protein) Data is provided in Supplementary Sheet *S4.xlsx* - Data Set S4.

4. The Lipidomics Data is provided in Supplementary Sheet *S5.xlsx* - Data Set 5.

Please browse the Full Text version of this manuscript to see the listed Data Sets.

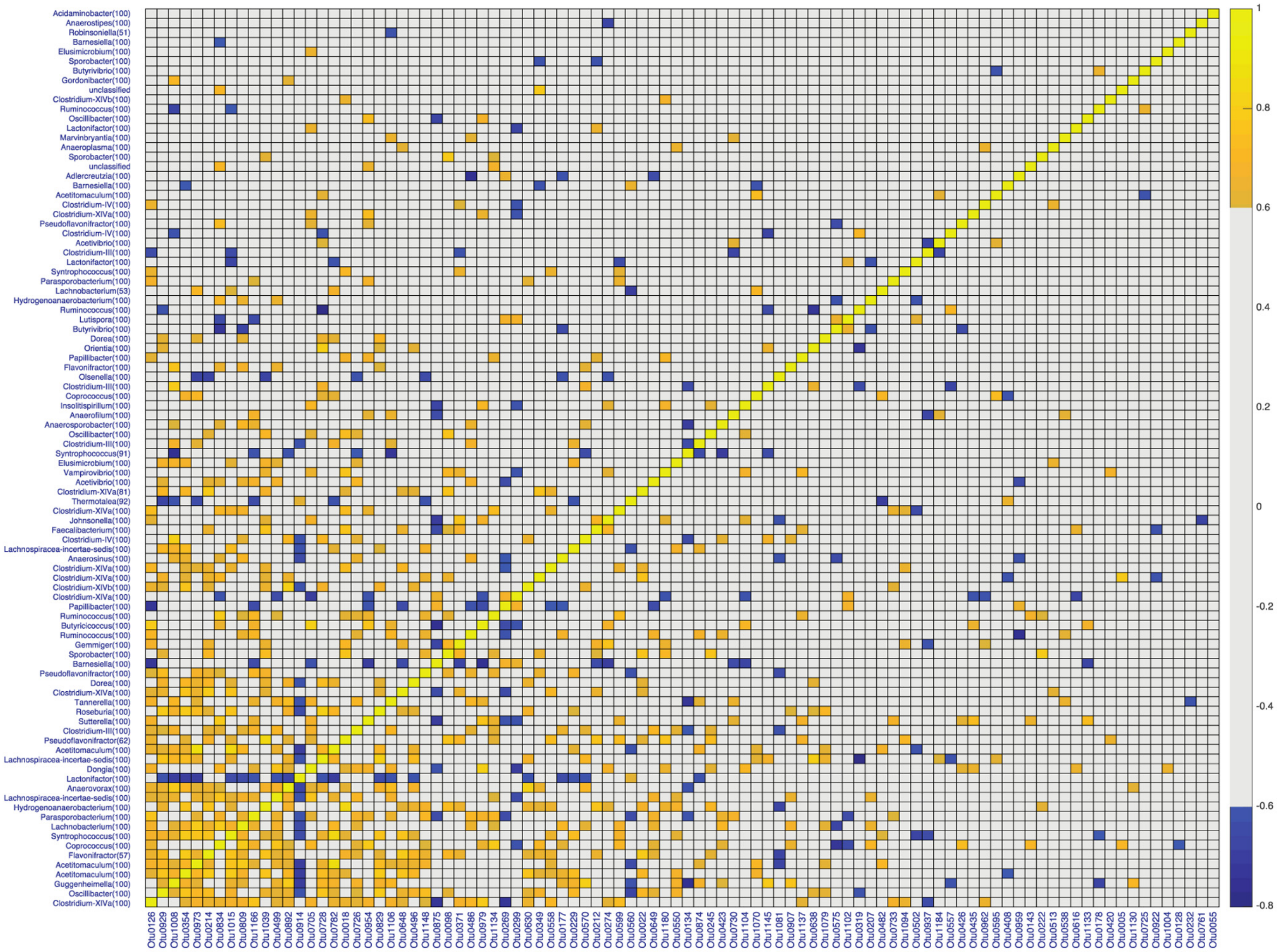

Supplementary Figure 1. Correlations between the statistically different OTUs across all aged rats. Yellow colors indicate positive correlations while blue colors indicate negative correlations. Grey boxes indicate either no correlations or correlations between -0.6 to 0.6 or non-statistically significant correlations. The rows have the OTUs classified at the genus level while the columns have the corresponding OTU IDs. It's a symmetric matrix thus column 1 corresponding to Otu0126 corresponds to row 1 and is classified as Clostridium XIVa. Correlations shown are after FDR correction with Q values < 0.05.

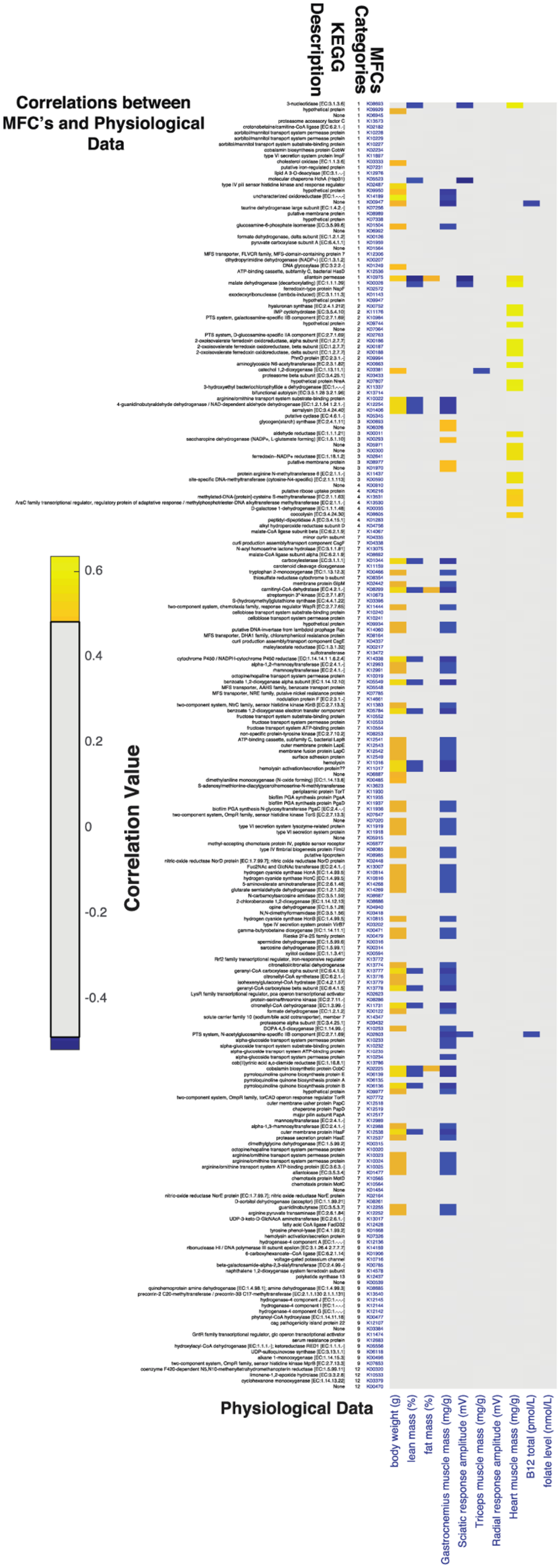

Supplementary Figure 2. Correlations between MFC's and physiological data. Yellow colors indicate positive correlations while blue colors indicate negative correlations. Grey boxes indicate either no correlations or correlations between -0.5 to 0.5 or non-statistically significant correlations. The rows correspond to the MFCs while the columns correspond to measured physiological data. Correlations shown are after FDR correction with Q values < 0.05.

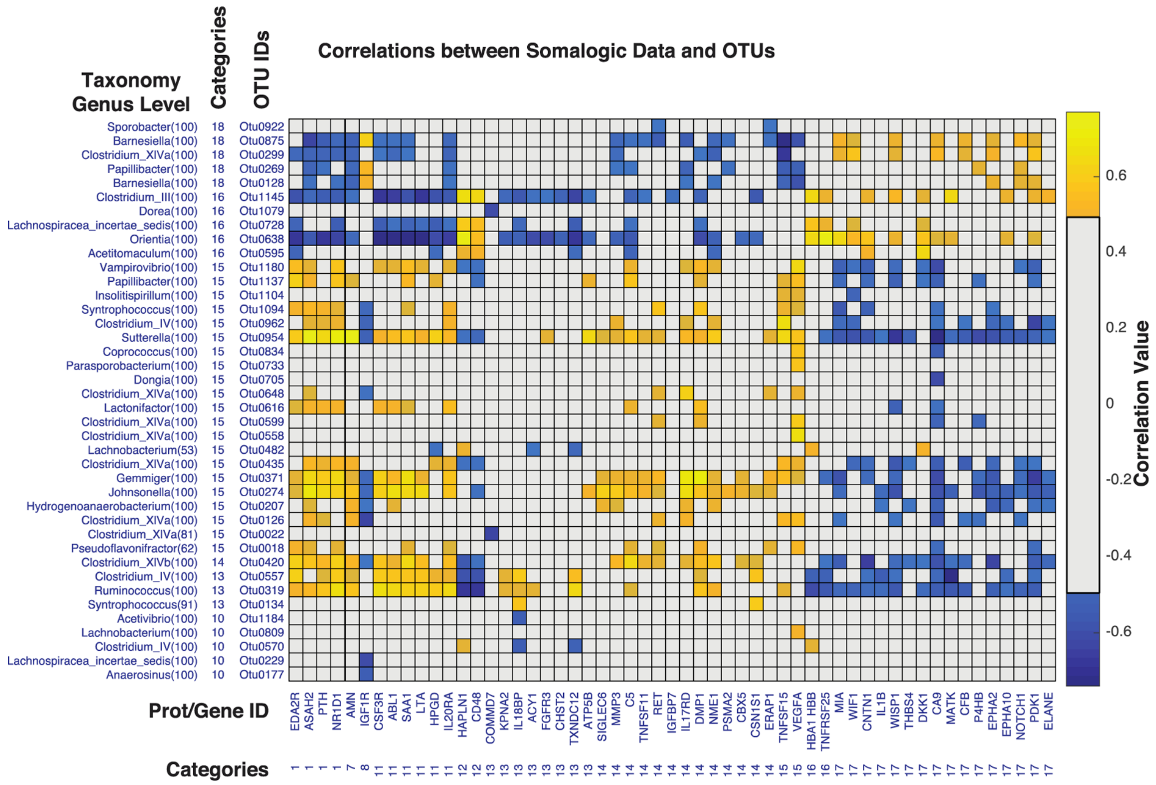

Supplementary Figure 3. Correlations between serum proteins and OTUs. Yellow colors indicate positive correlations while blue colors indicate negative correlations. Grey boxes indicate either no correlations or correlations between -0.5 to 0.5 or non-statistically significant correlations. The rows correspond to the OTUs while the columns correspond to serum proteins. Correlations shown are after FDR correction with Q values < 0.05.

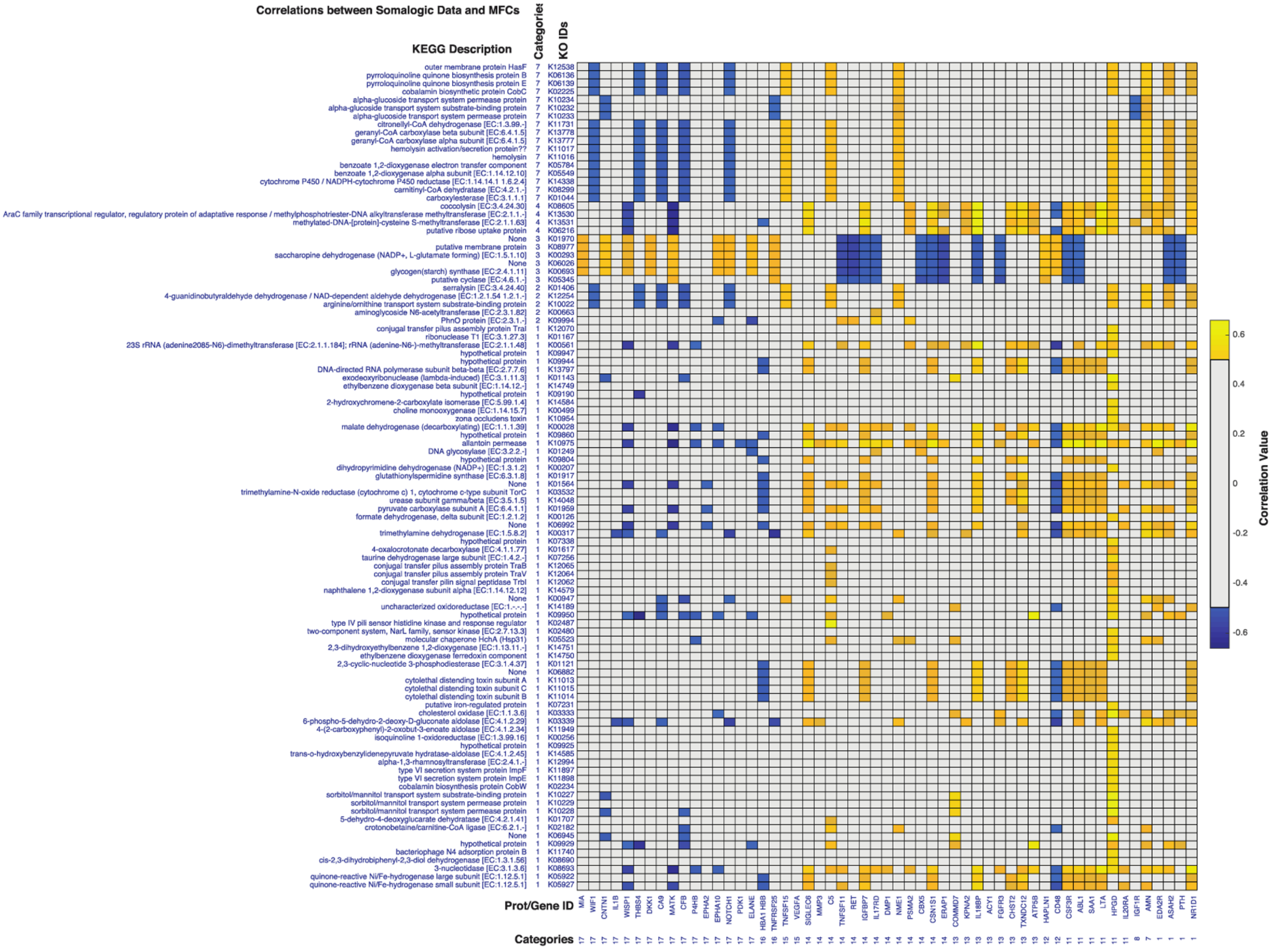

Supplementary Figure 4. Correlations between serum proteins and MFC's. Only statistically significant correlations are shown. Yellow colors indicate positive correlations while blue colors indicate negative correlations. Grey boxes indicate either no correlations or correlations between -0.5 and 0.5 or non-statistically significant correlations. Categorizations of MFC's and KEGG descriptions of the MFC's are also tabulated. Correlations shown are after FDR correction with Q values < 0.05.

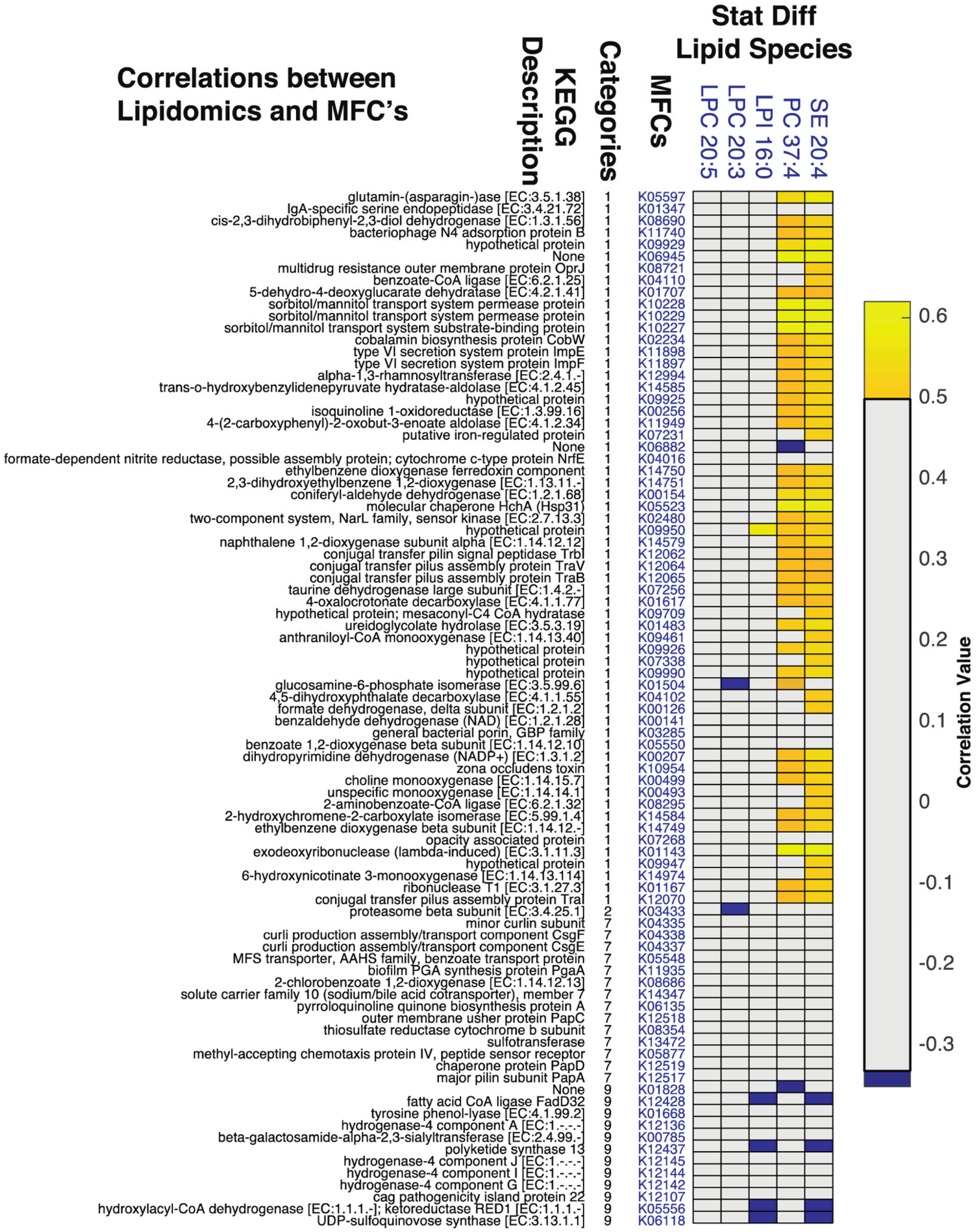

Supplementary Figure 5. Correlations between MFCs and lipids. Yellow colors indicate positive correlations while blue colors indicate negative correlations. Grey boxes indicate either no correlations or correlations between -0.35 to 0.5 or non-statistically significant correlations. The rows correspond to the MFCs while the columns correspond to lipids. Correlations shown are after FDR correction with Q values < 0.05.
